# The Practical Care of the Baby

**Published:** 1904-02

**Authors:** 


The Practical Care of the Baby. By Theron Wendell Kilmer, M. D., Associate Professor of Diseases of Children in the New York School of Clinical Medicine; Assistant Physician to the Out-Patient Department of the Babies' Hospital, New York; Attending Physician to the Children's Department of the West Side German Dispensary, New York. 12mo. Pages xiv-158, with 68 illustrations. Extra cloth, $1.00 net, delivered. Philadelphia: F. A. Davis Company, 1914-16 Cherry Street, Publishers.

This little book will be of benefit to the mother, and doubtless will save many a baby, if its suggestions are heeded. The doctor, too, will learn a thing or two from it, for many doctors (tell it not in Gath) are somewhat deficient in baby lore and some in common, practical sense. It is worth a dollar, dead easy.

